# Interferon-lambda (*IFNL*) germline variations and their significance for HCC and PDAC progression: an analysis of The Cancer Genome Atlas (TCGA) data

**DOI:** 10.1186/s12885-020-07589-4

**Published:** 2020-11-23

**Authors:** Henriette Huschka, Sabine Mihm

**Affiliations:** Department of Gastroenterology, Gastrointestinal Oncology, and Endocrinology, University Medical Center Goettingen, Robert-Koch-Str. 40, 37075 Goettingen, Germany

**Keywords:** *Interferon-lambda4 (IFNL4)*, *IFNL4* rs368234815, Type III interferons, Hepatocellular carcinoma (HCC), Pancreatic ductal adenocarcinoma (PDAC), Antitumor host response, Progression free interval (PFI), Overall survival (OS)

## Abstract

**Background:**

Hepatocellular carcinoma (HCC) and pancreatic ductal adenocarcinoma (PDAC) are malignancies with a leading lethality. With reference to interferons (IFNs) known to mediate antitumor activities, this study investigated the relationship between germline genetic variations in type III *IFN* genes and cancer disease progression from The Cancer Genome Atlas (TCGA) data. The genetic variations under study tag a gain-or-loss-of-function dinucleotide polymorphism within the *IFNL4* gene, rs368234815 [TT/ΔG].

**Methods:**

The entirety of the TCGA sequencing data was used to assess genotypes of 187 patients with HCC and of 162 patients with PDAC matched for ethnicity. Stratified for *IFNL* genotypes, both cohorts were subjected to time-to-event analyses according to Kaplan-Meier with regard to the length of the specific progression free interval (PFI) and the overall survival (OS) time as two clinical endpoints for disease progression.

**Results:**

Logrank analysis revealed a significant relationship between *IFNL* genotypes and disease outcome for PDAC. This relationship was not found for HCC. A multiple Cox regression analysis employing patients’ age, tumor grade and tumor stage as further covariates proved *IFNL* genotypes to be independent predictors for PDAC disease outcome.

**Conclusion:**

This repository-based approach unveiled clinical evidence suggestive for an impact of *IFNL* germline variations for PDAC progression with an *IFNL* haplotype predisposing for *IFNL4* expression being favorable.

## Background

Hepatocellular carcinoma (HCC) and pancreatic ductal adenocarcinoma (PDAC) are leading causes of cancer-related deaths [1]; they belong to the most lethal malignancies with 5-yr survival rates < 20% [2, 3]. According to Hanahan and Weinberg, dysregulated cellular pathways that transform growth of normal cells into neoplasms are regarded as ‘hallmarks of cancer’ [4]. The evasion of the host’s immune defense, later on, has been recognized as one further principle of promoting malignant growth [4]. The impact of the host’s immune system for disease progression is underscored by the recent successful clinical translation of immunotherapeutic strategies [5].

Understanding that not all patients benefit from cancer immunotherapy, the term ‘cancer immune responsiveness’ (CIR) has been coined [6]. Germline genetic variants have been proposed to contribute to CIR including those in IFN signaling genes [6]. IFNs and their effectors have been known for long not only to mediate antiviral but also to edit antitumor host responses [7, 8]. They divide into type I (IFN-α_n_/β), type II (IFN-γ) and type III (IFN-λ_1–4_). Among the genes encoding IFN species, only one, type III *IFNL4*, harbors a common exonic gain-or-loss-of-function variation: while the phylogenetically older variant ΔG enables functional IFN-λ_4_ protein expression, the knockout variant TT causes a frameshift thereby disrupting the open reading frame and preventing translation [9, 10]. This germline dinucleotide polymorphism, *IFNL4* rs368234815 [TT/ΔG] (merged into *IFNL4* rs11322783) thus reflects the ongoing process of pseudogenization dividing human beings into those who are predisposed to express IFN-λ_4_ protein and into those who are not [11]; it associates with clearance of hepatitis C virus (HCV) and a variety of other disease conditions (reviewed in [12]).

In the context of viral infections, generally, an IFN-λ_4_ creating genetic background rather was found to be unfavorable for the host. This counter-intuitive relationship was first recognized for HCV infection, when the IFN-λ_4_ creating genotypes were shown to be in LD with those *IFNL* variants that had been identified before to be correlated with poor clearance of HCV infection in genome wide association studies (GWAS) on main ethnic populations [10, 13]. Similarly, in human immunodeficiency virus (HIV) infection, the IFN-λ_4_ creating genotype was found to be associated with a higher prevalence of AIDS [14] while the non-encoding genotype associates with a lower probability to acquire HIV [15, 16]. Also cytomegalovirus (CMV) reactivation is described to be more prevalent in patients encoding for a functional IFN-λ_4_ protein [17, 18].

The availability of collaborative comprehensive data repositories enables analyses of patient material on a whole genome scale and on large sample sizes. The Cancer Genome Atlas (TCGA) database provides datasets on more than 11,000 cancer patients across 33 tumor entities to the scientific community. Besides demographic and clinical data, TCGA comprises whole exome DNA and RNA sequencing data not only of tissue samples derived from primary tumors but also from corresponding non-malignant material, the latter giving rise to patients’ germline genetic background.

By employing TCGA datasets, this study aimed at finding clinical evidence for or against an impact of *IFNL* germline variations for HCC or PDAC progression. Using the Kaplan-Meier estimator, disease progression was assessed by (i) the length of the specific progression-free interval (PFI) and by (ii) the overall survival (OS) time as two clinical outcome endpoints. A multivariate Cox proportional-hazards model was applied considering patients’ age, tumor grade, and tumor stage as covariates along with *IFNL* genotypes.

## Methods

### TCGA data portal

Analyses are based upon data generated by TCGA (phs000178.v10.p8) which is managed by the NCI and NHGRI. Specifically, projects on HCC (TCGA-LIHC; https://portal.gdc.cancer.gov/projects/TCGA-LIHC) and on PDAC (TCGA-PAAD; https://portal.gdc.cancer.gov/projects/TCGA-PAAD) were included. The access to controlled datasets was approved by NIH (project ID 20041). Open access demographic (gender, age at diagnosis, race and ethnicity) and clinical data (tumor grade and stage, specific PFI, OS time) were gathered from a curated and standardized dataset named TCGA Pan-Cancer Clinical Data Resource (TCGA-CDR) with a focus on clinical outcome endpoints [19].

### Reading-out *IFNL* genotypes

**Fig. 1:**
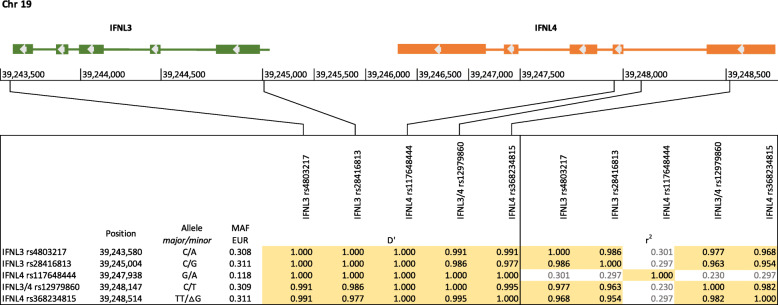
Localization of *IFNL* gene polymorphisms under study and calculation of LD. The 5 *IFNL* gene polymorphisms of interest are located within the 5′ and 3′ termini of the *IFNL3* gene and within exon 1 and 2 and intron 1 of *IFNL4* gene on chromosome 19 as shown. The normalized coefficients of LD (D’) and the correlation coefficients (r^2^) were calculated on data provided by the 1000 Genomes Project for individuals of European ancestry (*n* = 503). With the exception of *IFNL4* rs117648444, they qualify as mutual tagSNPs

Controlled access whole exome sequencing (WXS) reads of non-malignant tissue (code 11A) or peripheral blood leucocyte (code 10A) DNA, or sequencing reads of non-malignant tissue RNA, were cut down to the region spanning the *IFNL* gene cluster (chr19: 39,230,000 - 39,300,000) by using the BAM slice tool before downloading. By using the NCBI genome workbench software, the truncated sequence files were aligned to chromosome 19 of the human genome reference assembly GRCh38.p12. Depending on the depth of coverage, genotypes of up to five nucleotide polymorphisms were read out (Fig. 1). According to established criteria, a coverage of 20–30 sequence reads was considered reasonably confident. For heterozygous calls, both alleles should have an allele-call score > 10 and the ratio of their scores should be < 3. Call rates for both, the HCC and the PDAC cohort under study, reached 100%. Identical genotypes were obtained irrespective of whether malignant or non-malignant material was analyzed, on a sample basis.

### Statistical analysis

Exact test for deviation from Hardy-Weinberg equilibrium (HWE) was performed using an online calculator (http://www.dr-petrek.eu/documents/HWE.xls). Linkage disequilibrium (LD) coefficients D’ and r^2^ were enquired from the Web-based application LDlink (https://ldlink.nci.nih.gov) which refers to data of the 1000 Genomes Project (Phase 3, Version 5), by using the LDmatrix tool.

All other statistical analyses including time-to-event analysis according to the Kaplan-Meier method, and uni- and multivariate Cox proportional-hazard models were performed in R version 3.5.2 [20]. *P*-values ≤0.05 were considered statistically significant.

## Results

### The TCGA sample

The TCGA database contains data on 377 patients with hepatocellular carcinoma (HCC, C22.0) and on 185 patients with pancreatic ductal adenocarcinoma (PDAC, C25.-). They are assigned as African American, Asian, Native American, White, or of unknown ancestry (in numbers: 17 (4.5%) / 161 (42.7%) / 2 (0.5%) / 187 (49.6%) / 10 (2.7%) and 7 (3.8%) / 11 (5.9%) / 0 (0%) / 162 (87.6%) / 5 (2.7%), respectively). Besides demographical parameters, TCGA provides comprehensive clinical documentation. For instance, potential risk factors for the development of HCC are specified, as are infections with hepatitis viruses (e.g. HBV, HCV), intoxications (e.g. ethanol consumption), metabolic conditions (e.g. fatty liver disease), or others, or combinations of those. A descriptive, closer view on the data expectedly revealed HBV infection and HBV/HCV co-infections to be more prevalent among Asian patients, while isolated HCV infections and non-viral etiologies predominate among white patients (data not shown). Furthermore, patients with HBV infections featured more advanced tumor grades, and patients with HBV/HCV co-infections presented higher tumor stages (data not shown). To avoid bias due to ethnicity-related environmental differences, these observations prompted us to confine to the largest possible homogenous sample, i.e. the TCGA white patients, which were *n* = 187 for HCC and *n* = 162 for PDAC.

Regarding etiology, HCC patients divide into those with no or unknown history of primary risk factors (*n* = 54), with non-viral risk factors (*n* = 43), with confirmed HBV (*n* = 42) or HCV (*n* = 31) infection or with HBV/HCV co-infections (*n* = 41) (data not shown). Most of them – aged 63.3 yrs. on average at diagnosis – presented with tumor grade G2 (55.7%) and tumor stage I (47.3%) (Tab. 1). Starting at the time of diagnosis, the length of the median observation period for disease progression in terms of the specific PFI was 12.0 mo. During this period, 99 patients faced an event while 88 were censored. Regarding OS time, the median observation period was 22.1 mo. During this period, 77 patients deceased while 110 being censored. Median specific PFI amounts up to 19.7 mo, median OS time was 45.9 mo (Tab. 1).

**Table 1:** Patient characteristics

	HCC (*n* = 187)	PDAC (*n* = 162)
**Age**, mean ± SD [years]	63.3 ± 13.8	65.4 ± 10.8
**Gender**, m/f [n]	105/82	92/70
^1^**Tumor grade** [n (%)]		
G1	34 (18.6)	28 (17.4)
G2	102 (55.7)	84 (52.2)
G3	46 (25.1)	47 (29.2)
G4	1 (0.5)	2 (1.2)
^2^**Tumor stage** [n (%)]		
I	80 (47.3)	20 (12.6)
II	44 (26.0)	130 (81.8)
III	41 (24.3)	4 (2.5)
IV	4 (2.4)	5 (3.1)
**Specific PFI**		
median observation period (mo)	12.0	12.1
event / censored [n(%)]	99 (52.9) / 88 (47.1)	93 (57.4) / 69 (42.6)
median specific PFI (mo)	19.7	16.2
**OS time**		
median observation period (mo)	22.1	15.3
event / censored [n(%)]	77 (41.2) / 110 (58,8)	86 (53.1) / 76 (46.9)
median OS (mo)	45.9	20.2
***IFNL3*** **genotypes**		
rs4803217 CC:CA:AA [n(%)]	79 (42.2): 89 (47.6): 19 (10.2)	–
rs28416813 CC:CG:GG [n(%)]	–	76 (46.9): 69 (42.6): 17 (10.5)
MAF	0.340	0.318
HWE	*p* = 0.40	*p* = 0.82

^1^ Data on tumor grade were available for 183 and 161 patients with HCC and PDAC, respectively

^2^ Data on tumor stage were available for 169 and 159 patients with HCC and PDAC, respectively

PDAC patients aged 65.4 yrs. on average at the time of diagnosis presented above all with tumor grade G2, too, but compared to the HCC cohort, with a more advanced tumor stage in the majority of cases. Details to median observation periods and events are listed in Table 1. The median OS time was 20.2 mo, half the less of that for the HCC cohort.

### Genotyping

Patients’ genotypes were raised from up to five polymorphic sites within the *IFNL* gene cluster by aligning whole exome DNA and RNA sequence reads from non-malignant material to a reference genome. Coverage at *IFNL4* rs368234815 was found to be insufficient for most of the HCC and PDAC samples. An LD analysis based on the data of the 1000 Genomes Project and adjusted for the European population demonstrates that all polymorphic sites under study are in a nearly complete LD to each other (Fig. 1). Due to similar minor allele frequencies (MAF), four of them qualify as mutual tagSNPs (Fig. 1). Based on sequencing coverage rates, *IFNL3* rs4803217 and *IFNL3* rs28416813 were chosen as surrogates for the gain-or-loss dinucleotide polymorphism *IFNL4* rs368234815 for HCC and PDAC patients, respectively.

For HCC patients, genotype distribution of the surrogate SNP *IFNL3* rs4803217 was 79 (42.2%): 89 (47.6%): 19 (10.2%) (CC:CA:AA), meeting HWE. With a MAF of 0.340 the A allele carriers (*n* = 108) are supposed to correspond to those encoding a functional IFN-λ_4_ protein, while the C allele homozygotes resemble the *IFNL4* knockouts (Tab. 1).

Surrogate *IFNL3* rs28416813 genotype distribution of PDAC patients was 76 (46.9%): 69 (42.6%): 17 (10.5%) (CC:CG:GG) and was also found to match HWE. With a MAF of 0.318, 86 patients who are G allele carriers are supposed to be capable of expressing IFN-λ_4_ protein (Tab. 1).

### Analysis of disease progression with regard to *IFNL* genotypes

The length of the specific PFI and the OS time were chosen as clinical endpoints for disease progression. By employing Kaplan-Meier analyses, both parameters were analyzed with regard to patients’ *IFNL* genotypes.

**Fig. 2:**
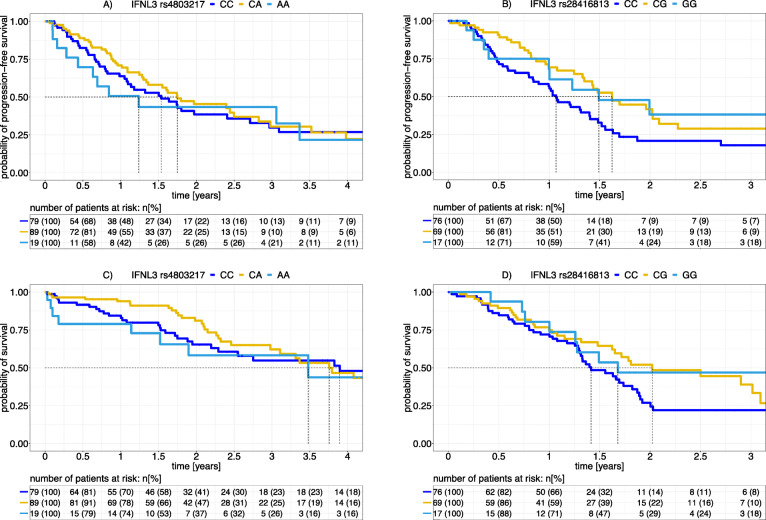
Time-to-event analyses for the length of the specific PFI and for OS time according to Kaplan-Meier for HCC and PDAC patients. HCC and PDAC patients were stratified for *IFNL3* rs4803217 and *IFNL3* rs28416813 genotypes, respectively. The probability of the absence of an event, which is progression (upper panels) or death (lower panels), is given in Kaplan-Meier graphs for each genotype for a period of 4 yrs. for HCC or 3 yrs. for PDAC as indicated. Dotted lines indicate the median specific PFIs and the median OS times. Tables list absolute and relative numbers of patients at risk (living and non-censored). A logrank test yielded a significant relationship between *IFNL* genotypes and disease outcome for PDAC patients (p(PFI) = 0.01, and p(OS) = 0.05, IFNL3 rs28416813 CC vs CG/GG) but not for HCC patients (p(PFI) = 0.65 and p(OS) = 0.87, IFNL3 rs4803217 CC vs CA/AA). This test was performed by comparing carriers of SNP variants that correspond to their ability to express IFNL4 (light blue and yellow) to knockout variant homozygotes (dark blue)

For HCC patients, the length of the median specific PFI did not relate to the number of the *IFNL3* rs4803217 alleles (gene dosage) (i.e., 18.4 mo: 21.0 mo: 14.9 mo for CC: CA: AA). The lack of this relationship became apparent also in Kaplan-Meier graphs (Fig. 2). The logrank test confirmed a lack of a significant difference in the length of the specific PFI between *IFNL3* rs4803217 C allele homozygotes and A allele carriers (*p* = 0.65). Similar non-significant results were obtained when the OS time as an endpoint of disease progression was analyzed with regard to *IFNL3* genotypes (*p* = 0.87, logrank test).

For PDAC patients, the length of the median specific PFI was shorter for *IFNL3* rs28416813 CC homozygotes than for G allele carriers (i.e., 12.8 mo: 19.5 mo: 17.9 mo for CC: CG: GG). This relationship manifests in the time-to-event Kaplan-Meier graphs (Fig. 2). The logrank test revealed a significant difference of the length of the specific PFI with CC homozygotes (corresponding to patients not encoding *IFNL4*) showing earlier disease progression than G allele carriers (*p* = 0.01). Similar results were obtained for OS time as a further clinical endpoint (*p* = 0.05, logrank test).

### Uni- and multivariate analyses of disease progression

In order to find out whether *IFNL* genotype is an independent parameter relating to disease progression, a multivariate Cox proportional-hazard model was applied. Parameters that revealed significant association in the univariate analysis were considered as covariates. These are patients’ age, tumor grade and tumor stage for HCC patients, and patients’ age, tumor grade, tumor stage and *IFNL* genotypes for PDAC patients, respectively.

For HCC, univariate analysis revealed tumor grade and tumor stage to be significantly related to the length of the specific PFI, while patients’ age was found to be significantly related to OS time (Tab. 2). Multivariate analysis revealed lower tumor grade compared to higher grade (G1 vs G2) tend to be independently associated with a twice as long specific PFI. A higher stage was found to be related to an up to 2-fold shortened specific PFI. Regarding OS time, multivariate analysis revealed patients’ age at diagnosis to be the only independent predictor (Tab. 2).

**Table 2:** Uni- and multivariate Cox proportional-hazard analyses for disease progression in HCC patients

Disease progression criteria	Univariate analysis		Multivariate analysis		Schoenfeld residuals
	HR (95% CI)	***p***-value	HR (95% CI)	***p***-value	***p***-value
^a^**Length of the specific PFI**					
Age	0.99 (0.98–1.00)	0.15	–	–	–
Gender (m/f)	0.87 (0.58–1.29)	0.48	–	–	–
Tumor grade (G1 vs. G2)	0.54 (0.30–0.97)	0.04*	0.54 (0.28–1.07)	0.08(*)	0.14
Tumor grade (G3/G4 vs. G2)	1.14 (0.73–1.80)	0.56	1.25 (0.78–2.02)	0.35	
Tumor stage (stage II vs. stage I)	1.78 (1.06–3.00)	0.03*	1.73 (1.01–2.93)	0.04*	0.02
Tumor stage (stage III/IV vs. stage I)	1.96 (1.19–3.24)	0.01*	2.12 (1.26–3.55)	< 0.01*	
*IFNL3* rs4803217 genotype (CA/AA vs. CC)	1.10 (0.74–1.63)	0.65	–	–	–
^a^**OS time**					
Age	1.03 (1.01–1.05)	0.02*	1.02 (1.00–1.04)	0.02*	0.93
Gender (m/f)	1.10 (0.70–1.73)	0.67	–	–	–
Tumor grade (G1 vs. G2)	0.50 (0.25–1.00)	0.05*	0.53 (0.27–1.07)	0.08(*)	0.25
Tumor grade (G3/G4 vs. G2)	0.91 (0.53–1.55)	0.72	1.01 (0.58–1.75)	0.96	
Tumor stage (stage II vs. stage I)	0.94 (0.50–1.79)	0.85	–	–	- -
Tumor stage (stage III/IV vs. stage I)	1.31 (0.75–2.26)	0.34	–	–	
*IFNL3* rs4803217 genotype (CA/AA vs. CC)	1.11 (0.71–0.74)	0.66	–	–	–

^a^ Cox regression analyses were performed for complete data sets (*n* = 167)

* significant p-value (*p* ≤ 0.5)

(*) tendency to significant *p*-value (*p* ≤ 0.1)

Results with a 95% CI including 1 and/or a significant Schoenfeld residual have to be considered with reservation.

For PDAC, univariate analyses similarly revealed tumor grade and tumor stage to be related to the length of the specific PFI, in addition to *IFNL* genotypes (Tab. 3). Multiple analysis proved tumor stage and *IFNL* genotypes to be independently and significantly related to disease progression in terms of the length of the specific PFI. Patients with tumor stage I face a 63% less probability for progression when compared to patients with stage II (*p* = 0.03). Patients with *IFNL* genotypes corresponding to the ability to express a functional IFN-λ_4_ protein had a 39% lower risk to face progression than patients with an *IFNL4* knockout haplotype (*p* = 0.02) (Tab. 3).

**Table 3:** Uni- and multivariate Cox proportional-hazard analyses for disease progression in PDAC patients

Disease progression criteria	Univariate analysis		Multivariate analysis		Schoenfeld residuals
	HR (95% CI)	***p***-value	HR (95% CI)	***p***-value	***p***-value
^a^**Lenghth of the specific PFI**					
Age	1.01 (0.99–1.03)	0.27	–	–	–
Gender (m/f)	1.13 (0.75–1.70)	0.56	–	–	–
Tumor grade (G1 vs. G2)	0.63 (0.33–1.20)	0.16	0.99 (0.51–1.90)	0.97	0.24
Tumor grade (G3/G4 vs. G2)	1.57 (1.01–2.45)	0.05*	1.34 (0.85–2.12)	0.20	
Tumor stage (stage I vs. stage II)	0.32 (0.14–0.74)	0.01*	0.37 (0.15–0.89)	0.03*	0.05
Tumor stage (stage III/ IV vs. stage II)	0.99 (0.46–2.16)	0.98	1.09 (0.50–2.39)	0.83	
IFNL3 rs28416813 genotype (CG/GG vs. CC)	0.60 (0.40–0.91)	0.02*	0.61 (0.40–0.93)	0.02*	0.60
^a^**OS time**					
Age	1.03 (1.01–1.05)	0.01*	1.01 (1.00–1.04)	0.13	0.67
Gender (m/f)	1.26 (0.83–1.93)	0.28	–	–	–
Tumor grade (G1 vs. G2)	0.76 (0.24–1.00)	0.05*	0.76 (0.36–1.60)	0.47	0.24
Tumor grade (G3/G4 vs. G2)	1.35 (0.85–2.13)	0.20	1.13 (0.70–1.81)	0.61	
Tumor stage (stage I vs. stage II)	0.37 (0.16–0.86)	0.02*	0.40 (0.16–1.04)	0.06(*)	0.01
Tumor stage (stage III/ IV vs. stage II)	0.90 (0.36–2.23)	0.82	0.95 (0.38–2.40)	0.92	
IFNL3 rs28416813 genotype (CG/GG vs. CC)	0.66 (0.43–1.00)	0.05*	0.68 (0.44–1.07)	0.09(*)	0.66

^a^ Cox regression analyses were performed for complete data sets (*n* = 158)

* significant p-value (*p* ≤ 0.5)

(*) tendency to significant *p*-value (*p* ≤ 0.1)

Results with a 95% CI including 1 and/or a significant Schoenfeld residual have to be considered with reservation.

Regarding OS time, patients’ age was found to be significantly related to this endpoint, too, in the univariate model. The multivariate model revealed patients with tumor stage II to face higher risk of mortality than patients with tumor stage I (*p* = 0.06), however, with reservations (Tab. 2). Multivariate analysis, moreover, yielded a tendency of an association for IFN-λ_4_ creating genotypes and a lower risk to decease (32%) when compared to *IFNL4* knockout haplotypes (*p* = 0.09).

## Discussion

Based on TCGA datasets, this study revealed significant associations between *IFNL* germline variations and progression of PDAC in terms of the length of the specific PFI and the OS time as two clinical endpoints. These *IFNL* variations are in nearly complete LD to a dinucleotide polymorphism that controls *IFNL4* gene expression (*IFNL4* rs368234815). By performing a multiple regression analysis including patients’ age at diagnosis, tumor stage, and tumor grade as further covariates, *IFNL* variation was proven to be an independent parameter for the length of the specific PFI (*p* = 0.02) and with a tendency to significance also for OS time (*p* = 0.09). This relationship was not observed for a cohort of patients matched for ethnicity but diagnosed for HCC.

A genetic background corresponding to the ability to express a functional IFN-λ_4_ protein, i.e. carriers of the *IFNL4* rs368234815 creating ΔG allele, was found to be related to a delayed progression of PDAC disease, i.e. being favorable. As outlined above, in the context of viral diseases, an IFN-λ_4_ creating genetic background – in general – is unfavorable for the host.

This disadvantage is also seen in the context of some cancer diseases, in particular for cancer entities with a virus related etiology. For instance, the *IFNL4* rs368234815 ΔG allele was shown to be associated with prostate cancer among men at increased risk of sexually transmitted infections [21]. In an independent study, this allele was shown to be related to significant decreased overall survival of African-American men with prostate cancer [22]. Moreover, susceptibility to AIDS-related Kaposi’s sarcoma was also found to be associated with genotypes predicted to produce an active IFN-λ_4_ [23].

In contrast, for PDAC, an entity for which no virus-related etiology is supposed, we found the genetic predisposition to encode for IFN-λ_4_ to be favorable for the outcome in terms of the length of the specific PFI and of the OS time. Even if biology of IFN-λ species is not yet completely understood, this is in accordance with the supposed general antitumor activity of type III IFNs [24, 25].

TCGA also provides information on cancer treatments. Where available, data comprise the type of the therapy, its starting date and duration, and the response to it. All of the HCC patients included into our analysis are documented to have received surgery, i.e. liver lobectomy or segmentectomy. Some of them received ablation (*n* = 40), adjuvant radiation (*n* = 7), or one or several regimens of chemotherapy (*n* = 12). Likewise, all of the PDAC cases under investigation were subjected to partial or total pancreatectomy. Some of them received adjuvant radiation therapy (*n* = 37) or adjuvant chemotherapy (*n* = 109). Our analyses were performed disregarding therapeutic schemes or their outcomes, which is a limitation. However, our analyses focusing on disease outcome in terms of the length of the specific PFI and OS time, are based on the assumption that patients were receiving the best possible care according to their individual health conditions. Nevertheless, the significant but weak association between *IFNL* genotype and clinical outcome for PDAC patients in the whole cohort might mask stronger associations within subgroups, e.g. among patients who are responding or non-responding to a cytostatic therapy. Data thus might reflect therapy responsiveness that, in turn, might translate into disease outcome. Accordingly, this association between *IFNL* variants and disease progression might be more prominent for PDAC than for HCC patients due to a higher proportion of patients subjected to chemotherapy, i.e. 109/162 (73.3%) vs 12/187 (6.4%), respectively. Whether treatment response is underlying the association between *IFNL* genotypes and cancer disease progression needs to be addressed in a separate analysis with a higher sample number. Alternatively, the lack of a relationship between *IFNL* genotypes and HCC progression might be related to that proportion of cases with viral etiology that distinguishes the HCC cohort from the PDAC cohort. The HCC cohort under study includes more than half of the patients (61%) with HBV infection (*n* = 42), with HCV infection (*n* = 31) or with HBV/HCV coinfections (*n* = 41).

## Conclusion

By employing a collaborative oncologic data repository with a given number of cases, TCGA facilitated the exploratory mining of clinical evidence suggestive for of an impact of *IFNL* germline variations on PDAC cancer progression. An *IFNL* haplotype predisposing for *IFNL4* gene expression appeared to be favorable for the host, which is in line with the concept of antitumor activities of type III IFNs. Further analyses will regard therapeutic interventions as additional variates.

## Data Availability

The raw datasets supporting the conclusions of this article are available via The Cancer Genome Atlas (TCGA) web portal (https://portal.gdc.cancer.gov/projects/TCGA-LIHC and https://portal.gdc.cancer.gov/ projects/TCGA-PAAD for HCC and PDAC datasets, respectively).

## Abbreviations

CIR: Cancer immune responsiveness
CMV: Cytomegalovirus
HBV: Hepatitis B virus
HCV: Hepatitis C virus
HCC: Hepatocellular carcinoma
HIV: Human immunodeficiency virus
HWE: Hardy-Weinberg equilibrium
IFN: Interferon
IFNL4: Interferon-lambda 4 gene
LD: linkage disequilibrium
MAF: Minor allele frequency
OS: Overall survival
PDAC: Pancreatic ductal adenocarcinoma
PFI: Progression free interval
SNP: Single nucleotide polymorphism
TCGA: The Cancer Genome Atlas
